# Supplementary material for: Higher CSF sTREM2 and microglia activation are associated with slower rates of beta‐amyloid accumulation
Source: EMBO Mol Med. 2020 Aug 10;12(9):e12308. doi: 10.15252/emmm.202012308 (PMC7507349; doi:10.15252/emmm.202012308)
Supplement: Supplementary file 1 — Expanded View Figures PDF [file EMMM-12-e12308-s001.pdf]

# Expanded View Figures

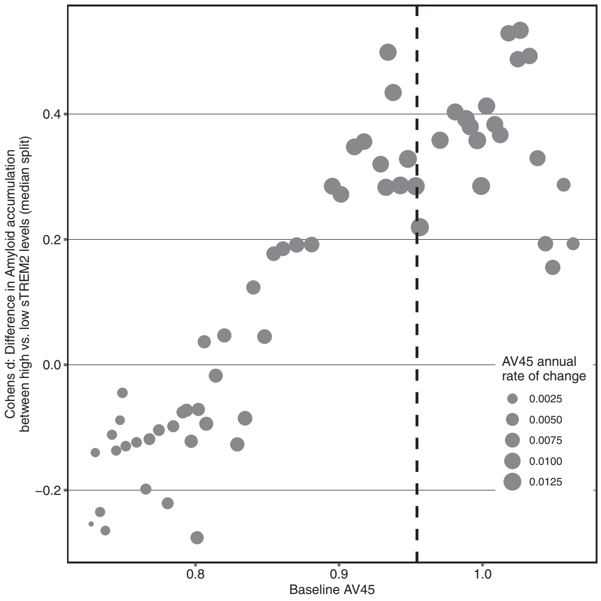


**Figure EV1. Sliding window analysis of sTREM2 effects on AV45 change rates.**

Sliding window analysis ($n$ = 100 per window, shifted in steps of 10) of sTREM2 effects on AV45 change rates at different levels of baseline AV45. For each window, subjects were divided into high vs. low sTREM2 via median split, and standardized group differences in AV45 change were determined as Cohen's *d*. The vertical dashed line marks the level of baseline AV45 at which subsequent AV45 changes are highest.

Source data are available online for this figure.

© 2020 The Authors
